# Romidepsin Promotes Osteogenic and Adipocytic Differentiation of Human Mesenchymal Stem Cells through Inhibition of Histondeacetylase Activity

**DOI:** 10.1155/2018/2379546

**Published:** 2018-03-14

**Authors:** Dalia Ali, Elna P. Chalisserry, Muthurangan Manikandan, Rimi Hamam, Musaad Alfayez, Moustapha Kassem, Abdullah Aldahmash, Nehad M. Alajez

**Affiliations:** ^1^Stem Cell Unit, Department of Anatomy, College of Medicine, King Saud University, Riyadh 11461, Saudi Arabia; ^2^Universite de Montreal, Departement de Medecine, Montreal, QC, Canada; ^3^Molecular Endocrinology Unit (KMEB), Department of Endocrinology, University Hospital of Odense and University of Southern Denmark, Odense, Denmark; ^4^Department of Cellular and Molecular Medicine, Danish Stem Cell Center (DanStem), University of Copenhagen, 2200 Copenhagen, Denmark; ^5^Prince Naif Health Research Center, King Saud University, Riyadh 11461, Saudi Arabia

## Abstract

Bone marrow mesenchymal stem cells (BMSCs) are adult multipotent stem cells that can differentiate into mesodermal lineage cells, including adipocytes and osteoblasts. However, the epigenetic mechanisms governing the lineage-specific commitment of BMSCs into adipocytes or osteoblasts are under investigation. Herein, we investigated the epigenetic effect of romidepsin, a small molecule dual inhibitor targeting HDAC1 and HDAC2 identified through an epigenetic library functional screen. BMSCs exposed to romidepsin (5 nM) exhibited enhanced adipocytic and osteoblastic differentiation. Global gene expression and signaling pathway analyses of differentially expressed genes revealed a strong enrichment of genes involved in adipogenesis and osteogenesis in romidepsin-treated BMSCs during induction into adipocytes or osteoblasts, respectively. Pharmacological inhibition of FAK signaling during adipogenesis or inhibition of FAK or TGF*β* signaling during osteogenesis diminished the biological effects of romidepsin on BMSCs. The results of chromatin immunoprecipitation combined with quantitative polymerase chain reaction indicated a significant increase in H3K9Ac epigenetic markers in the promoter regions of peroxisome proliferator-activated receptor gamma (PPAR*γ*) and KLF15 (related to adipogenesis) or SP7 (Osterix) and alkaline phosphatase (ALP) (related to osteogenesis) in romidepsin-treated BMSCs. Our data indicated that romidepsin is a novel in vitro modulator of adipocytic and osteoblastic differentiation of BMSCs.

## 1. Introduction

Epigenetic modulation refers to heritable changes in cellular gene expression via mechanisms that do not alter underlying DNA sequences. One epigenetic mechanism is the modulation of chromatin structure through histone modifications, including acetylation, methylation, and phosphorylation. Histone acetylation is regulated by the balance of opposing activities between histone acetyltransferases (HATs) and histone deacetylases (HDACs), which are important for the activation of gene transcription [1]. Chromatin architecture is an important factor in the regulation of gene expression because, through the process of active transcription, the compacted DNA is made accessible to RNA transcriptional machinery via epigenetic modification of the nucleosome. The changes in chromatin are affected via posttranslation modifications of histones; acetylation of core histones is one of the most studied types of modification implicated in regulating gene expression and cellular differentiation [2].

Because of their ability to modify histones, histone deacetylase inhibitors (HDACi) induce and regulate cell differentiation and modulate tissue-specific gene expression. In embryonic stem cells, HDACi reduce self-renewal by accelerating the expression of differentiation markers [3]. Previous studies indicated that HDACi enhanced the differentiation of human dental pulp stem cells into osteoblasts [4] and the differentiation of 3T3-L1 cells into adipocytes [5]. Valproic acid is an HDACi reported to regulate the proliferation and self-renewal of hematopoietic stem cells [6]. Glemzaite and Navakauskiene [7] investigated the epigenetic changes occurring during the differentiation of amniotic fluid-derived hMSCs into osteoblasts. The authors reported that HDAC1 and HDAC2 were downregulated, which are associated with an increase in H3K4me3 and H3K9Ac and which are associated with an active chromatin state [7].

Bone marrow stromal stem cells (BMSCs) (also known as skeletal or mesenchymal stem cells) are adult multipotent stem cells with the capacity to differentiate into mesodermal lineage cells, including adipocytes and osteoblasts [8]. To identify the molecular mechanisms underlying their lineage fate, we performed an epigenetic-library screen and identified a number of small molecule chemicals that suggested possible molecular mechanisms. For example, treatment of human BMSCs (hBMSCs) with the HDACi, abexinostat, induced significant changes in adipocytic and osteoblastic differentiation [9], and CUDC-907, a small molecule dual inhibitor of HDAC and PI3K, enhanced adipocytic differentiation [10]. Herein, we investigated the biological effects of romidepsin, a small molecule dual inhibitor of HDAC1 and HDAC2 [11], on the adipocytic and osteoblastic differentiation of hBMSCs and identified its molecular mechanism.

## 2. Materials and Methods

We followed the methods of Ali et al. [9].

### 2.1. Compound

Romidepsin was purchased from Selleckchem (Selleckchem Inc., Houston, TX, USA). Prior to use, romidepsin was dissolved in DMSO and used at a concentration of 5 nM. Control cells were treated with DMSO as the vehicle.

### 2.2. Cell Culture

We used a telomerized hBMSC line (hMSC-TERT) as a model for BMSCs. The hMSC-TERT line was created by overexpressing the human telomerase reverse transcriptase gene (hTERT). hMSC-TERT expresses all known markers of primary hBMSCs and exhibits “stemness” characteristics owing to its ability to form a bone and bone marrow microenvironment when implanted subcutaneously in vivo [12]. These cells are hereafter referred to as hBMSCs. Cells were cultured in a basal culture medium of Dulbecco's Modified Eagle's medium (DMEM), supplemented with 4500 mg/L D-glucose, 4 mM L-glutamine, 110 mg/L 10% sodium pyruvate, 10% fetal bovine serum (FBS), 1% penicillin-streptomycin, and 1% nonessential amino acids. All reagents were purchased from Thermo Fisher Scientific Life Sciences (Waltham, MA, USA, http://www.thermofisher.com). Cells were incubated in 5% CO_2_ incubators at 37°C and 95% humidity. The cells were cultured to reach 80–90% confluence before addition of romidepsin. Romidepsin was added at a concentration of 5 nM for 24 h. Thereafter, the cells were exposed to adipogenic or osteoblastic induction. Control cells were treated with basal medium containing dimethyl sulfoxide (DMSO) as a vehicle. Normal human primary hBMSCs were purchased from Thermo Fisher Scientific Life Sciences.

### 2.3. Adipogenic Differentiation

The adipogenic induction medium (AIM) consisted of DMEM supplemented with 10% FBS, 10% horse serum (Sigma-Aldrich, St. Louis, MO, USA, https://www.sigmaaldrich.com), 1% penicillin/streptomycin, 100 nM dexamethasone, 0.45 mM isobutyl methyl xanthine (Sigma-Aldrich), 3 mg/mL insulin (Sigma-Aldrich), and 1 mM rosiglitazone (BRL49653). The AIM was replaced every 3 days. Cells were assessed for adipogenic differentiation on day 7.

### 2.4. Oil Red O and Nile Red Staining

Adipogenic differentiation was determined by qualitative Oil Red O staining for lipid-filled mature adipocytes. Cells were washed with phosphate-buffered saline (PBS), fixed with 4% paraformaldehyde for 10 min, then incubated with freshly made and filtered (0.45 mM) Oil Red O staining solution (0.05 g in 60% isopropanol; Sigma-Aldrich) for 1 h at room temperature. Nile red fluorescence staining and quantification of adipogenesis were performed using a stock solution of Nile red (1 mg/mL) in DMSO that was stored at −20°C and protected from light. Staining was performed on unfixed cells. Cultured differentiated cells were grown in polystyrene flat-bottom 96-well tissue culture- (TC-) treated black microplates (Corning Inc., Corning, NY, USA, http://www.corning.com) and washed once with PBS. The dye was then added directly to the cells at a final concentration of 5 *μ*g/mL in PBS, and the preparation was incubated for 10 min at room temperature, then washed twice with PBS. The fluorescent signal was measured using a SpectraMax/M5 fluorescence spectrophotometer plate reader (Molecular Devices Co., Sunnyvale, CA, USA, https://www.moleculardevices.com) using the bottom well-scan mode, during which nine readings were taken per well using excitation (485 nm) and emission (572 nm) spectra. Oil Red and Nile red fluorescence were imaged using an EVOS Cell Imaging System (Thermo Fisher Scientific Life Sciences).

### 2.5. Osteogenic Differentiation

hBMSCs were cultured as noted in the previous section and exposed to osteogenic induction medium (DMEM containing 10% FBS, 1% penicillin-streptomycin, 50 mg/mL L-ascorbic acid (Wako Chemicals GmbH, Neuss, Germany, https://www.wakochemicals.de/), 10 mM *β*-glycerophosphate (Sigma-Aldrich), 10 nM calcitriol (1a, 25-dihydroxy vitamin D3; Sigma-Aldrich), and 100 nM dexamethasone (Sigma-Aldrich)).

### 2.6. Alkaline Phosphatase (ALP) Staining and Activity Quantification

We used a BioVision ALP activity colorimetric assay kit (BioVision, Inc., Milpitas, CA, USA, https://www.biovision.com/) with some modifications. Cells were cultured in 96-well plates under normal or osteogenic induction conditions. On day 10, wells were rinsed once with PBS and fixed using 3.7% formaldehyde in 90% ethanol for 30 s at room temperature. The fixative was removed, and 50 *μ*L of p-nitrophenyl phosphate solution was added to each well. The plates were incubated for 20–30 min in the dark at room temperature until a clear yellow color developed. The reaction was subsequently stopped by adding 20 *μ*L of stop solution. Optical density was then measured at 405 nm using a SpectraMax/M5 fluorescence spectrophotometer plate reader. For ALP staining, the cells were washed in PBS, fixed in acetone/citrate buffer, and incubated with ALP substrate solution (naphthol AS-TR phosphate 0.1 M Tris buffer, pH 9.0) for 1 h at room temperature. Images were taken using an EVOS Cell Imaging System (Thermo Fisher Scientific Life Sciences).

### 2.7. RNA Extraction and cDNA Synthesis

The total RNA was isolated from cell pellets after 7 days of adipogenic differentiation and 10 days of osteogenic differentiation using a Total RNA Purification Kit (Norgen Biotek Corp., Thorold, ON, Canada, https://norgenbiotek.com/) according to the manufacturer's protocol. The total RNA concentration was measured using a NanoDrop 2000 (Thermo Fisher Scientific Life Sciences). cDNA was synthesized using 500 ng of total RNA and the Thermo Fisher Scientific Life Sciences High Capacity cDNA Transcription Kit according to the manufacturer's protocol.

### 2.8. Quantitative Real-Time PCR

The expression levels of adipogenic-related genes and validation of selected upregulated genes in the microarray data (AP2, AdipoQ, PPAR*γ*2, CEBP*α*, PCK1, CNTFR, LPL, LIPE, and ACACB) were quantified using Fast SYBR Green Master Mix and a ViiA 7 Real-Time PCR device (Thermo Fisher Scientific Life Sciences). The primers used for gene expression analysis and validation are listed in Table 1. For osteoblast-related gene expression, custom TaqMan low-density array cards were used (Thermo Fisher Scientific Life Sciences). The assay ID for the primer sets used for the osteoblast gene panel is provided in supplementary Table 1. The 2DCT value method was used to calculate relative expression, and data was analyzed as previously described [13].

### 2.9. DNA Microarray Gene Expression Profiling

The total RNA (150 ng) was labeled using the low input Quick Amp Labeling Kit (Agilent Technologies, Santa Clara, CA, USA), then hybridized to the Agilent SurePrint G3 Human GE 8 × 60 k microarray chip (Agilent Technologies). All microarray experiments were performed at the Microarray Core Facility (Stem Cell Unit, King Saud University College of Medicine, Riyadh, Saudi Arabia). The extracted data were normalized and analyzed using GeneSpring 13.0 software (Agilent Technologies). Pathway analysis was performed using the Single Experiment Pathway analysis feature in GeneSpring 13.0 (Agilent Technologies) as previously described [14]. Twofold cutoff and a *P* < 0.05 were used to enrich for significantly changed transcripts.

### 2.10. Western Blotting

Total cellular protein was extracted using RIPA lysis solution (Norgen Biotek Corp., Thorold, ON, Canada). The protein (10 *μ*g) was resolved using Mini-PROTRAN®TGX™ Stain Free precast gels and transferred to a PVDF membrane using a Trans-Blot® Turbo™ Mini PVDF Transfer Pack (Bio-Rad Laboratories, Hercules, CA, USA). Blots were incubated with primary antibodies overnight at 4°C in TBS-Tween (0.05%) with 5% nonfat milk at the designated dilution for H3 (Lys9) (C5B11) rabbit monoclonal antibody (mAb) (1 : 1000; catalog number 9649, Cell Signaling Technology, Danvers, MA, USA, https://www.cellsignal.com) and di-methyl-histone H3 (Lys4) (C64G9) rabbit mAb (1 : 1000; catalog number 9725, Cell Signaling Technology). The membrane was subsequently incubated with anti-rabbit IgG HRP conjugated antibody (1 : 3000 dilution, Cell Signaling Technology, #7074p2). Membranes were probed using HRP-conjugated anti-glyceraldehyde-3-phosphate dehydrogenase (GAPDH) antibody (1 : 10,000, ab9482; Abcam, Cambridge, MA, USA, http://www.abcam.com) as a loading control. Imaging was conducted using the ChemiDoc MP imager (Bio-Rad Laboratories). Band intensity was quantified using the band quantification tool in the Image Laboratory 5.0 software (Bio-Rad Laboratories). Data were presented as the fold increase of normalized (to GAPDH) signal intensity of romidepsin-treated cells compared with DMSO-treated cells.

### 2.11. Chromatin Immunoprecipitation and qPCR Validation

hMSC-TERT cells (vehicle (DMSO) treated or romidepsin 24 h treated) were fixed with 1% formaldehyde for 15 min and quenched with 0.125 M glycine. Chromatin was isolated by adding lysis buffer and disrupting with a Dounce homogenizer. Lysates were sonicated, and the DNA was sheared to an average length of 300–500 base pairs. Genomic DNA (input) was prepared by treating aliquots of chromatin with RNase, proteinase K, and heat for decrosslinking, followed by ethanol precipitation. Pellets were resuspended, and the resulting DNA was quantified using a NanoDrop spectrophotometer. Extrapolation to the original chromatin volume allowed quantification of the total chromatin yield. An aliquot of chromatin (30 *μ*g) was precleared using protein A agarose beads (Invitrogen). Genomic DNA regions of interest were isolated using antibodies against H3K9Ac. Complexes were washed, eluted from the beads with SDS buffer, and subjected to RNase and proteinase K treatment. Crosslinks were reversed by overnight incubation at 65°C, and ChIP DNA was purified by phenol-chloroform extraction and ethanol precipitation. For quality assurance, quantitative PCR (qPCR) reactions were carried out in triplicate on specific genomic regions using SYBR Green Supermix (Bio-Rad Laboratories). The resulting signals were normalized for primer efficiency by performing qPCR for each primer pair using input DNA. All ChIP-seq experiments were performed by the Active Motif Epigenetic Service (Active Motif, Carlsbad, CA, USA).

### 2.12. HDAC Enzymatic Activity Assay

HDAC enzymatic activity in control or treated hMSC-TERT cells was measured using the HDAC-Glo I/II assay and screening system (Promega Inc., Madison, WI, USA, https://www.promega.com/) according to the manufacturer's protocol. Briefly, 1 × 10^4^ cells in a volume of 50 *μ*L were seeded per well in a white-walled 96-well plate and incubated with the inhibitor mixture at 37°C for 30 min. Trichostatin A was used as a positive control (supplied with the kit). HDAC-Glo I/II reagent (containing the substrate and the developer reagent) was added, and the solution was incubated at room temperature for 45 min. Luminescence was measured using a SpectraMax/M5 fluorescence spectrophotometer plate reader.

### 2.13. Inhibition of FAK and Transforming Growth Factor *β* (TGF*β*) Signaling during Adipogenic and Osteogenic Differentiation

hBMSCs were cultured in 96-well plates. After exposure to romidepsin or vehicle control for 24 h, normal culture medium was replaced with adipogenic and osteogenic induction medium supplemented with FAK inhibitor (PF-573228) at 5 *μ*M (Sigma-Aldrich) or transforming growth factor *β* (TGF*β*) signaling inhibitor (SB505124) at 1 *μ*M (Sigma-Aldrich). Adipogenic and osteogenic media were supplemented with inhibitors and replaced every 2 days. On day 7, Nile red was quantified to assess adipogenic differentiation, and on day 10, ALP was quantified to determine osteogenic differentiation, as indicated above.

## 3. Results

### 3.1. Romidepsin Promoted Adipocytic Differentiation of hBMSCs

Romidepsin is an HDACi that primarily targets HDAC1 and HDAC2. The compound was initially identified through a functional screen of an epigenetic library consisting of 24 compounds based on its ability to promote adipocytic and osteocytic differentiation of hBMSCs, as previously described [9].

**Figure 1:**
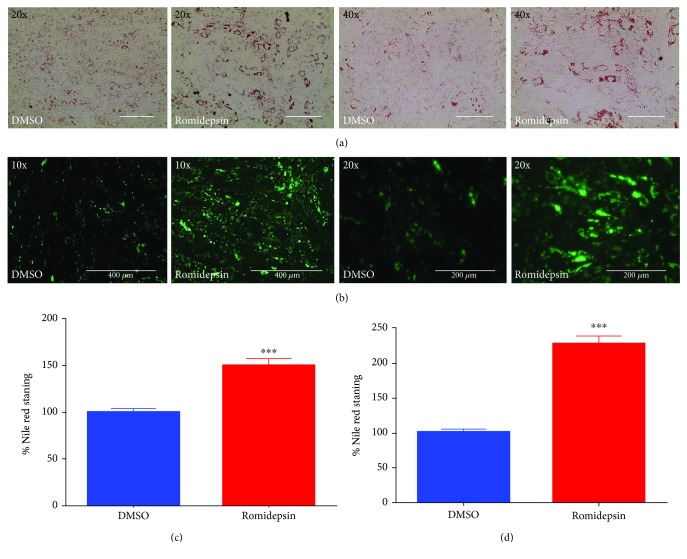
Effects of romidepsin treatment on the differentiation of human bone marrow stromal stem cells (hMSCs) into adipocytes. hMSCs were induced to differentiate into adipocytes in the presence of romidepsin (5 nM) or vehicle control for 24 h. The effects on adipocyte differentiation were examined at day 7. (a) Representative Oil Red O staining of lipid-filled mature adipocytes; (b) Nile red staining images were captured at ×20 and ×40 magnification using an EVOS Cell Imaging System; (c) the level of Nile red staining was quantified, and data are representative of three independent experiments; (d) Nile red staining quantification in primary hBMSCs. Data are presented as mean ± SEM, *n* = 15, ^∗∗∗^ *P* < 0.0005 from three independent experiments.

To investigate the mechanism by which romidepsin promotes bone marrow adipogenesis, hBMSCs were incubated with romidepsin (5 nM) for 24 h, followed by induction of adipocyte differentiation. hBMSC s exposed to romidepsin exhibited enhanced adipocyte differentiation, as indicated by higher Oil Red O staining (Figure 1(a)). Qualitative and quantitative analyses of mature adipocytes using Nile red staining (Figure 1(b)) and quantification (Figure 1(c)) revealed a significant increase (~1.5-fold, *P* < 0.0005; Figure 1(c)) in adipogenesis. Nile red quantification indicated that romidepsin promoted adipocyte differentiation in primary normal hBMSCs in a similar manner (~2.5-fold increase, *P* < 0.0005; Figure 1(d)).

### 3.2. Romidepsin Enhanced Adipocytic Gene Networks

**Figure 2:**
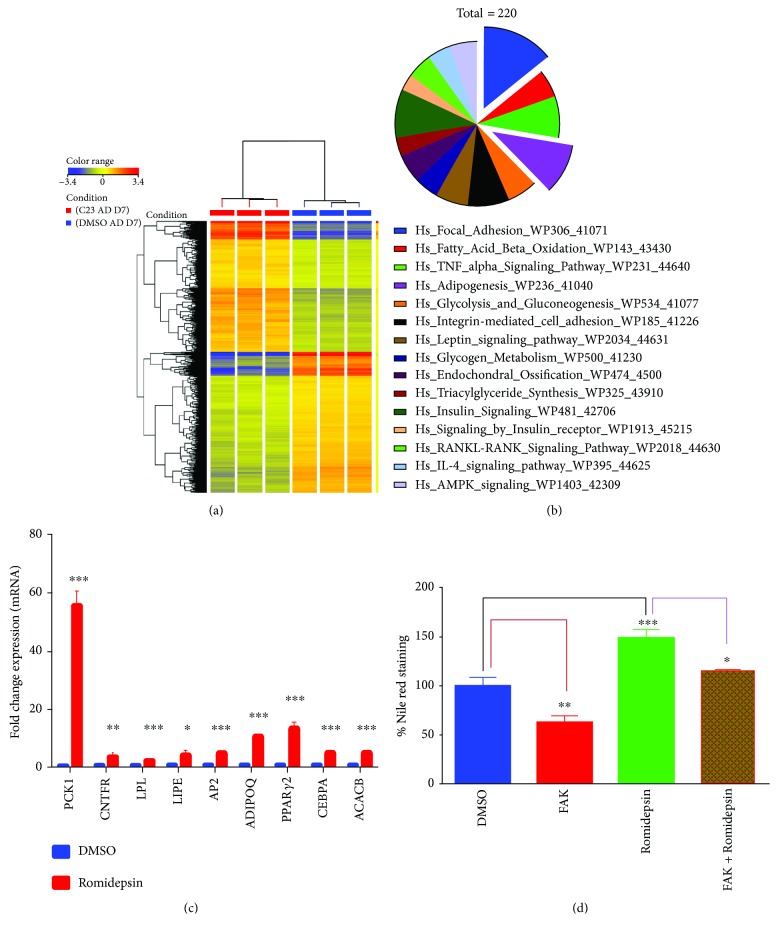
Microarray gene expression profiling of adipocyte-differentiated human bone marrow stromal stem cells (hMSCs) following romidepsin treatment. (a) Heat map and unsupervised hierarchical clustering were performed on differentially expressed genes in romidepsin-treated hMSCs compared to those in vehicle-treated control hMSCs on day 7 after adipocyte differentiation. (b) Pie chart illustrating the distribution of the top 15 enriched pathway categories in the differentially expressed genes identified in romidepsin-treated hMSCs. (c) Validation of a selected group of upregulated genes identified by microarray analysis using qRT-PCR. Gene expression was normalized to *β*-actin. Data are presented as mean fold changes ± SEM compared with vehicle-treated controls; *n* = 6 from two independent experiments, ^∗^ *P* < 0.05 and ^∗∗∗^ *P* < 0.0005 comparing romidepsin-treated and control cells. (d) Quantification of Nile red staining for mature adipocytes in romidepsin-treated hMSCs and in the absence or presence of a FAK inhibitor (5 *μ*M). Data are presented as mean ± SEM, *n* = 6, ^∗∗^ *P* < 0.005, and ^∗∗∗^ *P* < 0.0005.

To understand the molecular process by which romidepsin promoted adipocytic differentiation, global gene expression profiling of hBMSCs exposed to romidepsin and induced into adipocytes for 7 days was determined. Hierarchical clustering based on differentially expressed transcripts showed clear separation of the romidepsin-treated and control cells (Figure 2(a)). We identified 794 upregulated and 852 downregulated transcripts (>2.0 FC, *P* (Corr) < 0.05; Supplementary Table 2). Pathway analysis of the differentially expressed genes revealed significant enrichment of genes associated with several cellular processes, including focal adhesion and adipogenesis. The pie chart in Figure 2(d) depicts the top 15 enriched pathways. A selected gene panel from the genes identified by microarray analysis data based on their involvement in adipogenesis-related processes, including AdipoQ, AP2, PPAR*γ*2, CEBP*α*, CNTFR, LPL, LIPE, ACACB, and PCK1, was chosen for validation using qRT-PCR (Figure 2(c)). Concordant with the microarray data, inhibition of the FAK pathway using PF-573228 significantly inhibited the stimulatory effects of romidepsin (*P* < 0.0005; Figure 2(d)).

### 3.3. Effect of Romidepsin on Osteoblastic Differentiation of hBMSCs

**Figure 3:**
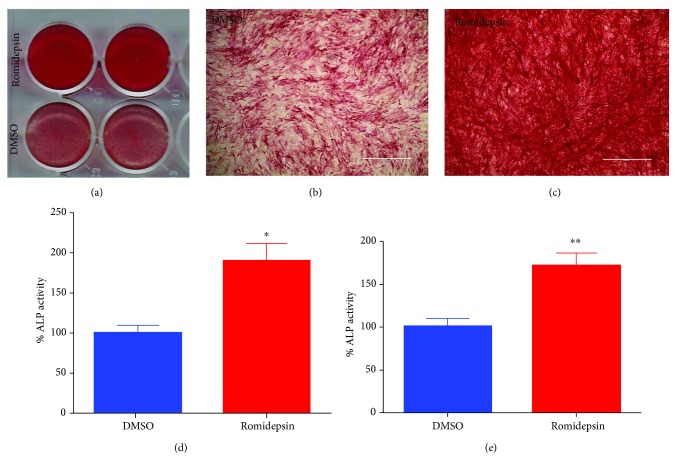
Effect of romidepsin treatment on the differentiation of human bone marrow stromal stem cells (hMSCs) into osteoblasts. hMSCs were induced to osteoblastic differentiation in the presence of romidepsin (5 nM) or vehicle control for 24 h. The effects on osteoblastic differentiation were examined at day 10. (a) ALP staining for romidepsin-treated compared to vehicle-treated control cells (4x magnification) using an EVOS cell imaging system. (b and c) Representative images of ALP staining from (a); (d) quantification of ALP activity; (e) validation of quantification of ALP activity in romidepsin-treated versus vehicle-treated control cells in primary hMSCs. Data are presented as mean ± SEM from two independent experiments, *n* = 15, ^∗^ *P* < 0.05, and ^∗∗^ *P* < 0.005.

We assessed the effect of romidepsin on the osteoblastic differentiation of hBMSCs. Cells were exposed to romidepsin for 24 h before exposing hBMSCs to osteoblastic induction media. On postinduction day 10, more ALP staining was observed in romidepsin-treated cells compared with vehicle-treated control cells (Figures 3(a)–3(c)). Similarly, ALP quantification revealed significantly higher ALP activity in the romidepsin-treated cells compared to that in the control cells (~2.0-fold increase, *P* < 0.05; Figure 3(d)). The stimulatory effect on osteoblasts was further validated using primary hBMSCs, which exhibited a significant increase in ALP activity in romidepsin-treated cells compared to that in control cells (~1.5-fold increase, *P* < 0.005).

### 3.4. Romidepsin Enhanced Osteoblastic Gene Networks

**Figure 4:**
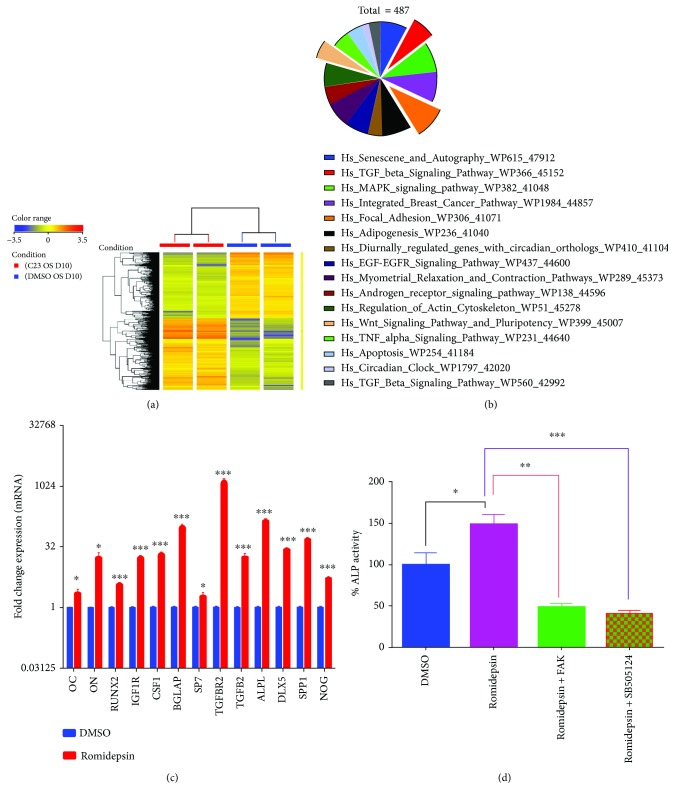
Microarray gene expression profiling of osteoblast-differentiated human bone marrow stromal stem cells (hMSCs) following romidepsin treatment. (a) Heat map and unsupervised hierarchical clustering were performed on differentially expressed genes induced by romidepsin compared to those of vehicle-treated control hMSCs at day 10 after osteoblastic differentiation; (b) Pie chart illustrating the distribution of the top 15 enriched pathway categories in the differentially expressed genes identified in romidepsin-treated hMSCs; (c) validation of the selected gene panel during osteoblastic differentiation using qRT-PCR. Gene expression was normalized to *β*-actin; (d) quantification of ALP activity in hMSCs induced to osteoblastic differentiation for 10 days after treatment with romidepsin in the absence or presence of a FAK inhibitor (PF-573228, 5 *μ*M) or TGF*β* inhibitor (SB505124, 5 *μ*M). Data are presented as mean ± SEM, *n* = 8; ^∗^ *P* < 0.05, ^∗∗^ *P* < 0.005, and ^∗∗∗^ *P* < 0.0005.

Global gene expression profiling was conducted on hBMSCs following exposure to romidepsin and osteoblastic induction for 10 days compared to that on vehicle-treated control cells. Hierarchical clustering based on differentially expressed transcripts showed clear separation between the romidepsin-treated and control cells (Figure 4(a)). We identified 3989 upregulated and 4315 downregulated transcripts (>2.0 FC, *P* (corr) < 0.05; Supplementary Table 3). Pathway analysis of the upregulated genes revealed strong enrichment for several cellular processes involved in osteoblastic differentiation, including focal adhesion, WNT signaling, and TGF*β* pathway (Figure 4(b)). We selected a group of genes from those that exhibited significant changes in the microarray analysis, based on their association with osteogenesis-related processes: ALP, OC, ON, RUNX2, IGF1R, CSF1, BGLAP, SP7, TGFBR2, TGFB2, ALPL, DLX5, SPP1, and NOG, using qRT-PCR (Figure 4(c)). Concordant with the microarray data, inhibition of FAK (using PF-573228) or TGF*β* (using SB505124) diminished the osteogenesis-promoting effects of romidepsin (*P* < 0.005, Figure 4(d)).

### 3.5. Romidepsin Promoted Adipogenesis and Osteogenesis via Inhibition of HDAC

**Figure 5:**
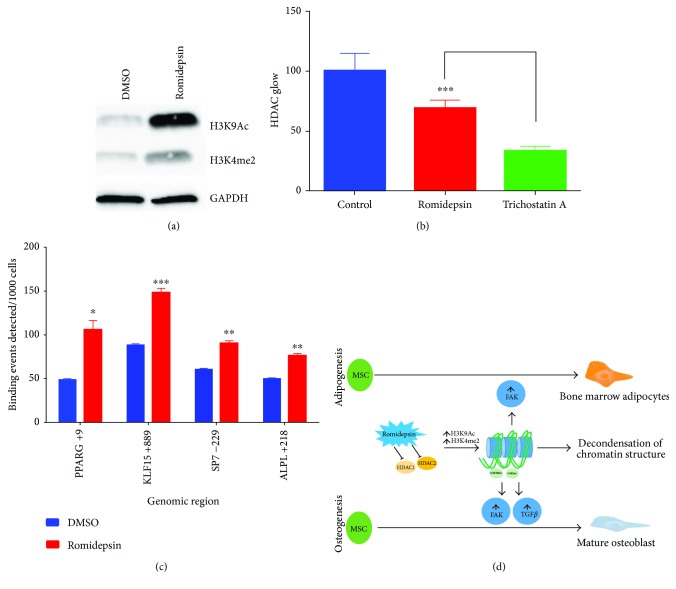
Romidepsin promoted adipogenesis and osteogenesis through inhibition of HDAC activity. (a) Western blot analysis of H3K9Ac and H3K4me2 histone markers in romidepsin-treated cells (24 h) versus vehicle-treated control cells. (b) Quantification of total cellular HDAC enzymatic activity of romidepsin-treated hMSCs (30 min) compared with control cells. Trichostatin A was used as a positive control. Data are presented as mean ± SEM from two independent experiments, *n* = 11, ^∗∗∗^ *P* < 0.0005. (c) H3K9Ac ChIP-qPCR for the promoter regions of PPARG, KLF15, SP7, and ALPL in romidepsin versus DMSO-treated hMSCs. Data are presented as mean binding events detected per 1000 cells ± SD (*n* = 3). (d) Working model of the molecular mechanisms of enhanced adipocytic and osteogenic differentiation of hMSCs by romidepsin through amplification of the FAK and TGF*β* signaling pathways. ^∗^ *P* < 0.05; ^∗∗^ *P* < 0.005.

To identify the molecular mechanism by which romidepsin promoted adipocytic and osteocytic differentiation, hBMSCs treated with romidepsin for 24 h were examined for different histone markers using Western blotting. The results indicated increased levels of H3K9Ac and H3K4me2, which are associated with actively transcribed genomic regions (Figure 5(a)). The increase in those epigenetic markers was associated with a significant decrease in HDAC activity (~70% reduction, *P* < 0.0005; Figure 5(b)). Trichostatin A- (TA-) treated cells were used as positive control (Figure 5(b)).

### 3.6. ChIP-qPCR Data Revealed Significant Enrichment in Multiple Genes Related to Adipogenesis and Osteogenesis

We subsequently sought to determine the promoter regions in hBMSCs targeted by romidepsin using CHIP assay with H3K9Ac antibody. The immune-precipitated genomic DNA was subjected to qPCR for the promoter regions of PPARG, KLF15 (related to adipogenesis) and SP7, ALPL (related to osteogenesis). The results indicated a significant increase in the H3K9Ac epigenetic marker in the promoter regions of these genes in response to romidepsin treatment, likely through the inhibition of HDAC1 and HDAC2 (Figure 5(c)).

## 4. Discussion

Understanding the molecular mechanism underlying the differentiation fate choice of hMSCs into osteoblasts and adipocytes is important for understanding disease processes in which hMSC differentiation is affected, for example, bone fragility in age-related osteoporosis and obesity [15]. In the current study, we report the use of a chemical biology approach using a known HDACi to uncover the relevance of epigenetic regulation in hBMSC differentiation. We identified a number of genes and genetic pathways that are relevant for both osteoblast and adipocyte differentiation.

In our studies, we employed a cell model, hMSC-TERT, for hBMSCs which has been created through overexpression of human telomerase reverse transcriptase gene (hTERT). The hMSC-TERT cells express known markers of primary hBMSCs, exhibit stemness characteristics, and are able to form bone and bone marrow microenvironment when implanted in vivo [12, 16]. Our previous gene expression profiling highlighted the similarities of the molecular phenotype of hMSC-TERT cells and primary hBMCs [17]. Previous data showed that HDAC inhibition reduces telomerase protein expression and activity which may confound the results obtained in our study. However, we obtained similar effects of romidepsin on osteoblastic and adipocytic differentiation in primary bone marrow MSCs (Figures 1(d) and 3(e)) obtained from normal healthy donor suggesting that the observed effects of romidepsin on hBMSC differentiation are not related to its effects on telomerase activity.

In the current study, we investigated romidepsin, an HDAC1 and HDAC2 inhibitor, that has been used as an anticancer drug and is known to induce cancer cell cycle arrest, cancer cell differentiation, and cell death [18]. We observed that hMSCs are more sensitive to romidepsin because the biological effects were observed at a much lower dose (5 nM) compared to the biologically effective dose of two small molecule epigenetic modifiers, abexinostat and CUDC-907, that we reported previously [9] [10]. We observed that romidepsin induced adipocytic differentiation through the upregulation of adipocyte-associated transcriptional factors known to be required for adipocyte differentiation, for example, peroxisome proliferator-activated receptor gamma 2 (PPAR*γ*2) and CCAAT/enhancer binding protein *α* (CEBP*α*) [19–21] and other genes relevant to adipogenesis, including ADIPOQ, AP2, PCK1, LPL, and LIPE. These genes are important for both adipocyte lineage commitment and maturation [22]. Among the upregulated genes, ACACB is part of the adipocytokine signaling pathway and CNTFR is known to prevent neuronal degeneration. Additionally, the expression of these genes has been implicated in adipogenesis leading to obesity and diabetes [23, 24]. We observed that approximately 48% of the upregulated genes in romidepsin-treated hMSCs that were induced to differentiate into adipocytes were also upregulated in either abexinostat- or CUDC-907-treated hMSCs, suggesting a common mechanism of action for these compounds.

Romidepsin treatment enhanced the differentiation of hBMSCs into osteoblasts through upregulation of a number of osteogenic gene markers, including ALP, OC, ON, and RUNX2 [25]. Insulin-like growth factor 1 receptor (IGF1R) signaling, which regulates Osterix (SP7), and RUNX2 are important factors in bone formation during development and in the postnatal organism [26–28]. In addition, another upregulated gene in romidepsin-treated cells was DLX5, which activates bone formation directly through activation of RUNX2 [29].

We reported that romidepsin treatment uncovered important genetic pathways that are common or specific to the differentiation of hBMSCs into osteoblasts and adipocytes. Romidepsin treatment enhanced the FAK intracellular signaling pathway known to regulate BMSC differentiation to osteogenic or adipogenic lineage [30, 31]. For example, Hu et al. reported that changes in cellular physical environment by shockwave treatment enhanced osteogenesis via activation of FAK signaling followed by activation of ERK1/2 and RUNX2 [32]. On the other hand, disruption of FAK signaling in the preadipocyte cell line, 3T3L1 cells, or in vivo in mice decreased adipocyte cell differentiation and impaired insulin sensitivity [33].

In addition to its effect on FAK signaling, romidepsin enhanced two osteoblast-associated signaling pathways: TGF*β* and Wnt signaling. TGF*β* is important for the proliferation, commitment, and differentiation of BMSCs to osteoblastic lineages directly and through cross-talk with other intracellular signaling pathways such as BMPs and Wnt [34]. The importance of TGF*β* isoforms and their receptors in skeletal biology and BMSC differentiation and functions has been demonstrated by the presence of significant skeletal defects in TGF*β* genetically modified animals; for example, TGF*β*2 knockout mice showed lack of distal parts of the ribs [35], transgenic mice with negative form of TGF*β*2 developed hypoplastic cartilage, multiple defects in the base of the skull and vertebrae, defects in long bones and joints [36], and important for cartilage maintenance and prevention of osteoarthritis in human and mice models [37].

Wnt signaling functions are a potent regulator of osteogenesis in hBMSC, and its inhibition is accompanied by inhibition of osteoblastic differentiation [38]. Gaur et al. reported that canonical Wnt signaling is significantly linked to RUNX2 which is a target for *β*-catenin/TCF1 nuclear translocation, a signaling pathway required for osteoblastic differentiation and bone formation [39]. We have previously demonstrated that hBMSCs carrying activation mutation of Lrp5 (Wnt coreceptor) lead to enhanced Wnt signaling and osteoblast differentiation but inhibit adipocyte differentiation [40].

Our data suggest that romidepsin magnified the effects of osteoblast and adipocyte induction media. We observed that following treatment with romidepsin, H3K9Ac histone marks were increased suggesting an active state of chromatin and increased the accessibility of transcription factors to their target genes, inducing osteogenic and adipocytic differentiation [41]. This model is supported by the ChIP-qPCR results and gene expression analysis that demonstrated a significant upregulation of Kruppel-like factors (KLFs). KLFs are a member of a family of C2H2 zinc finger proteins known to regulate adipocyte differentiation [42]. KLF15 has been reported to synergistically interact with CEBP*α* to induce adipogenesis via increasing the activity of PPAR*γ*2 and CEBP*α*. In addition, PPAR*γ*2 induces the adipogenic differentiation program with induction of adipogenic genes, such as AP2 and Adipo Q. While these effects are known to take place in extramedullary adipogenic differentiation [43], our data suggest similar effects on bone marrow adipocytes. For osteoblastic cell differentiation and mineralized matrix formation, the coordinated effects of bone-specific transcriptional factors, SP7 (Osterix) and RUNX2, are needed [44]. Our ChIP-qPCR corroborated these concepts as it revealed a significant increase in the H3K9Ac marks at the SP7 promoter region.  Figure 5(d) presents our current working hypothesis regarding the interaction of romidepsin and HDAC inhibition in hBMSC lineage fate determination.

## 5. Conclusions

The study of the epigenetic pathways that modulate the adipogenic and osteogenic differentiation of hBMSCs provides useful information and insight into the understanding of the cross-talk between the epigenetic effect of HDACi, transcription factors, and differentiation pathways. The ability to manipulate intracellular signaling pathways associated with hBMSC differentiation state can optimize the use of hBMSCs in clinical applications and regenerative medicine.

## Figures and Tables

**Table 1:** List of SYBR green primers used in current study.

No.	Name	Sequence
1	AdipoQ	F 5′ GCAGTCTGTGGTTCTGATTCCATAC
		R 5′ GCCCTTGAGTCGTGGTTTCC
2	AP2	F 5′ TGGTTGATTTTCCATCCCAT
		R 5′ GCCAGGAATTTGACGAAGTC
3	CEBPA	F 5′ TATAGGCTGGGCTTCCCCTT
		R 5′ AGCTTTCTGGTGTGACTCGG
4	PPAR*γ*2	F 5′ TTCTCCTATTGACCCAGAAAGC
		R 5′ CTCCACTTTGATTGCACTTTGG
5	PCK1	F 5′ TTACCAATGTGGCCGAGACC
		R 5′ GCACAAGGTTCCCCATCCTC
6	CNTFR	F 5′ GCGACTCTAGCCTTGTCACC
		R 5′ GACTGTGTCTCTGGGCGTAG
7	LPL	F 5′ CTTGGAGATGTGGACCAGC
		R 5′ GTGCCATACAGAGAAATCTC
8	LIPE	F 5′ AGCCACGATGGGTGGAATG
		R 5′ ACCAGCGACTGTGTCATTGT
9	ACACB	F 5′ ACAGTCCTGAGATCCCCCTC
		R 5′ GTTCAGCCGGGTGGACTTTA
10	*β*-actin	F 5′ AGCCATGTACGTTGCTA
		R 5′ AGTCCGCCTAGAAGCA

## Supplementary Materials

Supplementary 1 Supplementary Table 1: list of TaqMan assay ID for the Osteo qPCR gene panel primers.

Supplementary 2 Supplementary Table 2: list of differentially expressed genes (2.0 FC, *P* corr < 0.05) in human mesenchymal stem cells (hMSCs) differentiated into adipocytes (day 7) in the presence of romidepsin compared to control hMSCs.

Supplementary 3 Supplementary Table 3: list of differentially expressed genes (2.0 FC, *P* corr < 0.05) in human mesenchymal stem cells (hMSCs) differentiated into osteoblasts (day 10) in the presence of romidepsin compared to control hMSCs.
