# Supplementary material for: Romidepsin Promotes Osteogenic and Adipocytic Differentiation of Human Mesenchymal Stem Cells through Inhibition of Histondeacetylase Activity
Source: Stem Cells Int. 2018 Mar 14;2018:2379546. doi: 10.1155/2018/2379546 (PMC5872662; doi:10.1155/2018/2379546)
Supplement: Supplementary 1 — Supplementary Table 1: list of TaqMan assay ID for the Osteo qPCR gene panel primers. [file 2379546.f1.docx]

| **Supplementary Table 1. List of Taqman assay ID for the Osteo qPCR gene panel primers** | | |
| --- | --- | --- |
| **No** | **Name** | **Assay ID** |
| 1 | ON | Hs00270274_m1 |
| 2 | RUNX2 | Hs01047973_m1 |
| 3 | IGF1R | Hs00609566_m1 |
| 4 | CSF1 | Hs00174164_m1 |
| 5 | OC | Hs01587814_g1 |
| 6 | SP7 | Hs01866874_s1 |
| 7 | TGFBR2 | Hs00234253_m1 |
| 8 | TGFB2 | Hs00234244_m1 |
| 9 | ALPL | Hs01029144_m1 |
| 10 | DLX5 | Hs00193291_m1 |
| 11 | SPP1 | Hs00959010_m1 |
| 12 | NOG | Hs00271352_s1 |
